# SDF-1α Gene-Activated Collagen Scaffold Restores Pro-Angiogenic Wound Healing Features in Human Diabetic Adipose-Derived Stem Cells

**DOI:** 10.3390/biomedicines9020160

**Published:** 2021-02-06

**Authors:** Ashang L. Laiva, Fergal J. O’Brien, Michael B. Keogh

**Affiliations:** 1 Tissue Engineering Research Group, Department of Anatomy and Regenerative Medicine, Royal College of Surgeons in Ireland, 123 St. Stephen’s Green, Dublin 2, Ireland; lluwang@rcsi-mub.com (A.L.L.); fjobrien@rcsi.ie (F.J.O.); 2 Department of Biomedical Science, Royal College of Surgeons in Ireland, Adliya, P.O. Box 15503 Manama, Bahrain; 3 Trinity Centre for Bioengineering, Trinity Biomedical Sciences Institute, Trinity College Dublin, Dublin 2, Ireland; 4 Advanced Materials and Bioengineering Research Centre, Royal College of Surgeons in Ireland and Trinity College Dublin, Dublin 2, Ireland

**Keywords:** gene-activated scaffold, SDF-1α, human diabetic ADSCs, angiogenesis, wound healing

## Abstract

Non-healing diabetic foot ulcers (DFUs) can lead to leg amputation in diabetic patients. Autologous stem cell therapy holds some potential to solve this problem; however, diabetic stem cells are relatively dysfunctional and restrictive in their wound healing abilities. This study sought to explore if a novel collagen–chondroitin sulfate (coll–CS) scaffold, functionalized with polyplex nanoparticles carrying the gene encoding for stromal-derived factor-1 alpha (SDF-1α gene-activated scaffold), can enhance the regenerative functionality of human diabetic adipose-derived stem cells (ADSCs). We assessed the impact of the gene-activated scaffold on diabetic ADSCs by comparing their response against healthy ADSCs cultured on a gene-free scaffold over two weeks. Overall, we found that the gene-activated scaffold could restore the pro-angiogenic regenerative response in the human diabetic ADSCs similar to the healthy ADSCs on the gene-free scaffold. Gene and protein expression analysis revealed that the gene-activated scaffold induced the overexpression of SDF-1α in diabetic ADSCs and engaged the receptor CXCR7, causing downstream β-arrestin signaling, as effectively as the transfected healthy ADSCs. The transfected diabetic ADSCs also exhibited pro-wound healing features characterized by active matrix remodeling of the provisional fibronectin matrix and basement membrane protein collagen IV. The gene-activated scaffold also induced a controlled pro-healing response in the healthy ADSCs by disabling early developmental factors signaling while promoting the expression of tissue remodeling components. Conclusively, we show that the SDF-1α gene-activated scaffold can overcome the deficiencies associated with diabetic ADSCs, paving the way for autologous stem cell therapies combined with novel biomaterials to treat DFUs.

## 1. Introduction

Stem cell-driven wound healing is an inherent biological process that occurs to restore a damaged tissue [1]. The stem cells are recruited in response to signals released from the wound site such as the stromal-derived factor-1 alpha (SDF-1α) and colony stimulating factor (CSF) [2,3]. However, in patients with underlying medical conditions such as diabetes, stem cells recruitment to the wound site is impaired, contributing to progression of the wound to a more deleterious chronic state [4,5]. In diabetic foot ulcer patients, poor prognosis poses a serious risk for amputation, quality of life and mortality of the patient [6]. The involvement of many factors such as vascular insufficiency, neuropathy, susceptibility to infection, local recurrence risk and the propensity to aggravate presents a major challenge in finding an ultimate treatment of the healing disorder [4].

Rapid healing is considered the key to prevent amputation in DFU patients [7]. Emerging evidence suggests that the application of tissue-engineered grafts significantly outperforms the standard of care (i.e., debridement and infection control with regular dressing changes) in the ability to accelerate healing [8]. Apligraf^®^ and Dermagraft^®^ are two of the widely used FDA-approved bioengineered constructs for diabetic foot ulcers [9]. Apligraf^®^ comprises a dermal layer of human neonatal fibroblasts in a bovine type I collagen matrix and an epidermal layer formed by human neonatal keratinocytes [10], while Dermagraft^®^ is generated by culturing human neonatal fibroblasts on a bioresorbable polyglactin scaffold [11]. However, these constructs are not approved for application over wounds with exposed muscle, tendon or bone [10]. Moreover, the proportion of patients responding effectively to these grafts is moderate (56% for Apligraf^®^ [11] and 30% for Dermagraft^®^ [12]. In particular, Apligraf^®^ is also known to show a relatively short persistence in the wound (~4 weeks), leading to the belief that the accelerated healing outcome is potentially due to the factors secreted by cells in Apligraf^®^ [13].

Stem cell therapy is an emerging solution for non-healing wounds. The efficacy of stem cell therapy is primarily attributed to stem cells’ self-renewal capacity, paracrine and immunomodulatory effects and the ability to differentiate and remodel the tissue matrix [14]. Stem cells from allogenic sources including bone marrow [15], adipose tissue [16] and the umbilical cord [17] have been used to treat DFU patients. One potential reason for using allogenic stem cells is the impaired functionality of autologous diabetic stem cells [18,19,20,21]. Therapeutic gene delivery to diabetic stem cells is one of the approaches that might potentially improve the functionality of diabetic stem cells [22]. Conventional gene delivery strategies involve relatively invasive intradermal injections [23,24]. Genes are often delivered in high amounts (<100 μg) with such strategies. Our previous studies have found that combining therapeutic genes with biomimetic collagen scaffolds (gene-activated scaffold) is an effective approach for enhancing tissue regeneration. The biomimetic scaffold acts a platform for supporting the three-dimensional growth of the cells while also facilitating their transfection [25,26]. The use of gene-activated scaffolds can promote a significant healing response with a genetic cargo dose as low as 2 μg [27]. The enhanced wound healing potency of gene-activated scaffolds is attributed to their ability to produce controlled spatiotemporal expression of the therapeutic transgene at the wound site [28].

**Figure 1:**
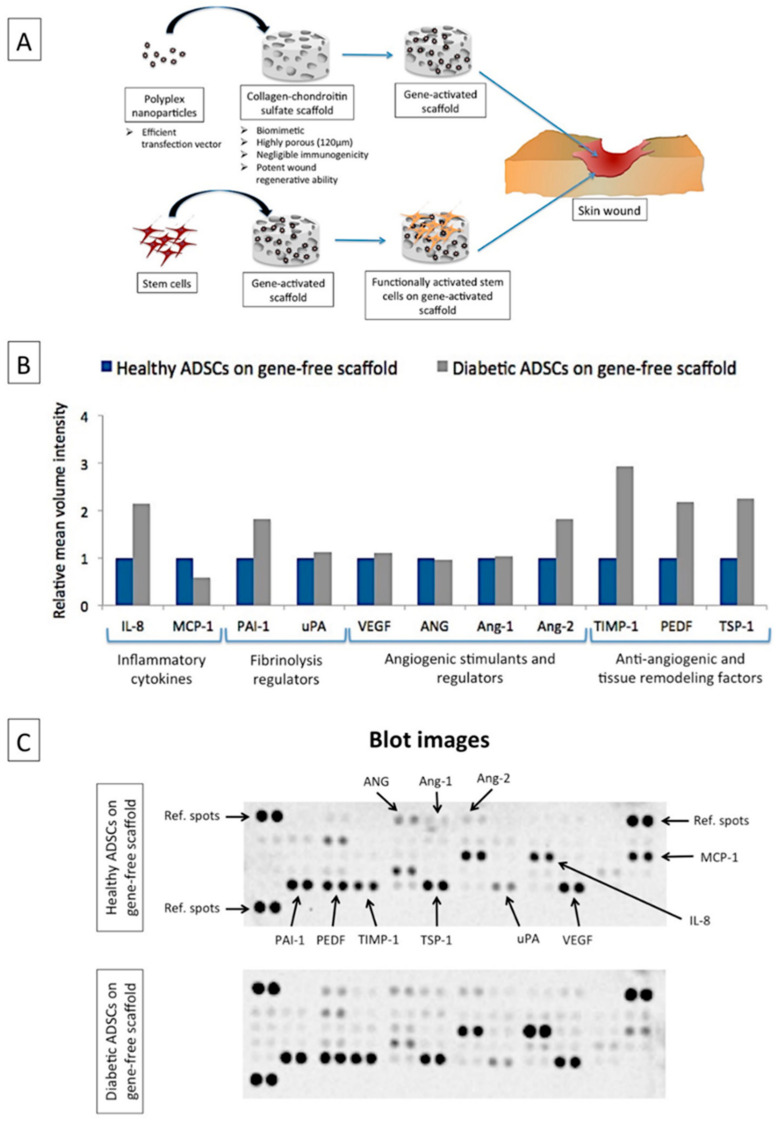
Gene-activated scaffold for wound healing applications and its impact on human adipose-derived stem cells (ADSCs). (**A**) A schematic of the application of a gene-activated scaffold for wound healing. Gene-activated scaffolds can be directly implanted into the wound where host cells infiltrating the gene-activated scaffold induce the reparative effects. Alternatively, the tissue engineering approach can be adopted where stem cells are grown on the gene-activated scaffold and the resulting construct is implanted into the wound site. (**B**) Relative expression of functional factors produced by human diabetic ADSCs on the gene-free scaffold compared to healthy ADSCs on the gene-free scaffold on day 7. An angiogenesis proteome profiler was used to screen the contents of the ADSCs secretome. Conditioned media (CM) were pooled in equal volumes from the three replicates to assay the proteome. Expression levels were quantified based on mean volume intensities determined using a ChemiDoc system. The diabetic ADSCs on the gene-free scaffold produced elevated levels of anti-angiogenic factors (PAI-1, TIMP-1, PEDF and TSP-1), inflammatory cytokine IL-8 and vascular disruptive factor Ang-2 relative to their healthy equivalent. (**C**) Representative blot images of the proteome analysis.

In our lab, we rely on the use of non-viral-based vectors such as polyethyleneimine (PEI) for the transfection of cells. PEI possesses high transfection efficiency over a range of cell types and can easily condense in the presence of plasmid DNA to form highly stable nanoparticles [28,29]. The process of developing a non-viral-based gene-activated scaffold first involves formulating nanoparticle complexes of a vector and a plasmid encoding the therapeutic gene. The nanoparticles are then soak-loaded onto the biomaterial scaffolds. These nanoparticles sit on the pore’s sidewalls in the scaffold, where they are taken up by the cells as the cells migrate through the pores [25,26]. Cellular uptake of the nanoparticles leads to functional activation of the cells, which eventually signals surrounding cells to stimulate local tissue regeneration [25,26]. Common biomimetic scaffolds used for the development of gene-activated scaffolds include collagen/chondroitin sulfate (coll–CS) [30] and/or hyaluronic acid [31] and collagen/chitosan [32] that are known to exhibit potent wound healing properties. A schematic of the development of a gene-activated scaffold is presented in Figure 1A. Multiple genes (e.g., bone morphogenic protein-2 and VEGF) can also be delivered through the gene-activated scaffold to further enhance stem cell differentiation and tissue repair in vivo [27]. Alternatively, the gene-activated scaffold can be functionalized with silencing RNAs (e.g., transforming growth factor- beta 1) to limit unwanted healing outcomes such as scarring in skin regeneration [33].

Stem cells from bone marrow (BM-MSCs) are often the cell candidates while developing gene-activated scaffold-based therapeutic modalities [34,35,36]. A recent study by Kolakshyapati et al. also showed that an epidermal growth factor gene-activated scaffold enhanced bone marrow stem cells’ differentiation into sweat gland-like cells, leading to the regeneration of sweat gland-like structures in vivo [36]. However, diabetic patients suffer from reduced stem cell populations in bone marrow [37], suggesting that BM-MSCs may not be ideal for developing personalized tissue-engineered products for diabetic patients [38]. Human umbilical cord blood stem cells are another attractive stem cell candidate. They have also been approved by the FDA but only for the treatment of blood-related disorders [39]. An example product is CLEVECORD^TM^ [40]. However, the stem cells yield in the cord blood is very low and their functionality is often limited by poor engraftment in the host tissue [39]. In this regard, adipose tissue represents a more desirable source for harvesting autologous stem cells. The yield capacity of stem cells from adipose tissue (ADSCs) could be as high as 500 times that of the stem cells derived from the same mass of bone marrow [41]. Moreover, the stem cells from adipose tissue could be harvested using a minimally invasive liposuction process [41]. Alofisel^®^ and Queencell^®^ are examples of ADSCs-based products approved for use in Europe and South Korea, respectively, for the treatment of Crohn’s disease [40]. Recently, ADSCs-loaded hydrogel sheets (ALLO-ASC-sheet; Anterogen, Seoul, South Korea) have also been shown to enhance healing in DFU patients [42].

Biomaterial scaffolds developed from the copolymer of type I collagen and chondroitin sulfate (coll–CS) are some of the most clinically efficacious scaffolds used for wound healing [30]; for example, Integra’s dermal regeneration template, which also received FDA approval for use in DFU treatment [43]. Our lab uses an optimized freeze-drying protocol to produce coll–CS scaffolds with highly uniform pore architectures, which facilitates efficient cell adhesion and infiltration within the scaffold [44]. Recently, we showed that coll–CS can be functionalized with nanoparticles carrying the pro-angiogenic gene SDF-1α (SDF-1α gene-activated scaffold) and significantly enhance the pro-angiogenic responses in BM-MSCs and human Schwann cells [34,45]. SDF-1α primarily functions as a chemokine to recruit endothelial progenitors at the wound site to promote angiogenesis [46]. However, it is deficient in diabetic wounds [46] and is known to localize predominantly at the wound margins [47]. Moreover, SDF-1α is also known to exert a pro-survival effect in ADSCs [48]. Therefore, we sought to investigate if the SDF-1α gene-activated scaffold could be used to restore the regenerative potential of human diabetic ADSCs and engineer a functionally enhanced graft for wound healing. Having initially established that diabetic stem cells are functionally impaired [18,19,20,21], we investigated the functional improvement in diabetic ADSCs with reference to healthy ADSCs cultured on the gene-free coll–CS scaffold. The functional improvement was determined based on the production of an array of angiogenic/anti-angiogenic factors, angiogenic bioactivity of the secreted factors and the expression patterns of extracellular matrix genes and proteins essential for wound healing.

## 2. Materials and Methods

### 2.1. Preparation of Polyplex

Plasmid DNA (pDNA) encoding for the therapeutic gene SDF-1α (pSDF-1α) was obtained from InvivoGen, San Diego, USA. The plasmids were first amplified by transforming chemically competent DH5α E. coli cells (Biosciences, Dublin, Ireland) according to the manufacturer’s protocol. Transformed cells were then expanded in Lysogeny broth (LB) plates containing 100 μg/mL of blasticidin as the selective antibiotic for pSDF-1α. After 24 h at 37 °C, bacterial colonies were harvested and amplified in LB broth containing the appropriate antibiotic and cultured overnight in a shaker incubator at 37 °C. Plasmid purification was performed using a QIAGEN^®^ EndoFree^®^Plasmid Maxi kit (Qiagen, Sussex, UK) and final nucleic acid concentration was determined using NanoDrop 1000 spectroscopy. Plasmids were further diluted in TE buffer to obtain a working concentration of 0.5 μg/μL and stored at −20 °C until use. Plasmid DNA (pDNA) encoding a non-therapeutic Gaussia luciferase (pLuc) purchased from New England Biolabs, Massachusetts, USA, was similarly amplified using ampicillin as the selective antibiotic. Based on our previous study [34], polyplex particles were formulated by initially mixing a specified amount of branched cationic 25 kDa PEI (Sigma-Aldrich, Wicklow, Ireland) and anionic pDNA (fixed at a dose of 2 μg) to give an N/P ratio of 10.

### 2.2. Cell Expansion

Human ADSCs (healthy, female, age 33, Cat no. 10HU-001, Lot no. 200359; diabetic type 2, female, age 45, Cat no.10HU-007, Lot no. 200404) purchased from iXCells Biotechnologies, were expanded to passage 4 in ADSCs growth medium (Cat no. MD0003) supplied by the company.

### 2.3. Cell Seeding on SDF-1α Gene-Activated Scaffold

Solid porous coll–CS scaffolds were first developed by freeze-drying a blend solution of collagen type I and chondroitin sulfate, using the optimized protocol developed in our lab [49,50]. The scaffolds were then dehydrothermally treated under vacuum at 105 °C and further crosslinked using 14 mM N-(3-Dimethylaminopropyl)-N′-ethylcarbodiimide hydrochloride and 5.5 mM N-Hydroxysuccinimide (EDAC/NHS) (Sigma, Gillingham, UK) to mechanically reinforce the scaffolds. Using these gene-free coll–CS scaffolds, a preliminary test group was created by culturing healthy or diabetic ADSCs. Briefly, the scaffolds were hydrated in PBS and placed in a 12-well plate. The ADSCs at a total density of 5 × 10^5^ cells (2.5 × 10^5^ per side) were then seeded onto the scaffolds. After letting the cells settle for about 20 min, 2 mL of OptiMEM (transfection media) was added, and the cellularized scaffolds were incubated at 37 °C for 24 h. For the final test group, an SDF-1α gene-activated scaffold was developed, which involved soak-loading the PEI-pSDF-1α polyplex nanoparticles into the freeze-dried scaffolds. Healthy/diabetic ADSCs at the same cell density as on the gene-free scaffold were seeded and incubated in OptiMEM. In this experimental run, healthy ADSCs on the gene-free scaffold were used as control. After incubation in OptiMEM for 24 h, the cellularized gene-free or gene-activated scaffolds were transferred into new 12-well plates and fed with 2 mL of ADSCs growth medium. Media change was then performed every 3–4 days until day 14 by collecting 1 mL of the conditioned media (CM) and replacing them with new media. All CM were stored at −80 °C until analysis.

### 2.4. Proteome Profiling of Secreted Factors from Healthy and Diabetic ADSCs on the Gene-Free Scaffold

In order to first confirm functional impairment in diabetic ADSCs, secreted factors produced by diabetic ADSCs were compared against those produced by healthy ADSCs, using an angiogenesis proteome profiler (ARY007, R&D Biosystems, UK). Based on the technique adopted in previous studies [51,52], equal volumes of CM pooled from each replicate (*n* = 3; 500 μL/group) on day 7 were used for the analysis. The amount of secreted factors was semi-quantitatively determined from the mean volume intensities obtained using ChemiDoc XRS+ (Biorad). The high-sensitivity mode of ChemiDoc XRS+ was used to detect the bound analytes on the array. Change in the expression of the protein was then determined relative to that of the healthy ADSCs on the gene-free scaffold.

### 2.5. qRT-PCR Analysis to Determine Functional Gene Expression in ADSCs on SDF-1α Gene-Activated Scaffold

In order to determine the activation of functional genes, the ADSCs from the scaffolds or SDF-1α GAS were harvested on the 7th and 14th days for analysis. The cells were first lysed using Qiazol reagent (Qiagen, Manchester, UK) to extract the RNA. Chloroform was then added to separate the cell lysate into protein, DNA and RNA phases. RNA was extracted using the RNeasy Kit (Qiagen, UK). The RNA quality and quantity were determined using a Multiskan Go plate reader (Thermo Scientific, Gloucester, UK) with the absorbance set at 260 nm. Prior to using a reverse transcriptase enzyme (Qiagen, UK) for cDNA synthesis, genomic DNA was removed by heating the RNA to 42 °C for 2 min using a genomic DNA wipeout buffer (Qiagen, UK). qRT-PCR was then performed on cDNA using the following primers: Hs_CXCL12_1_SG, Hs_CXCR4_1_SG, Hs_ACKR3_1_SG, Hs_ARRB_1_SG, Hs_FN1_1_SG and Hs_COL4A1_1_SG, which encode for SDF-1α, CXCR4, CXCR7, β-arrestin, fibronectin and collagen IV, respectively. Fold change in mRNA expression relative to the respective controls at days 7 and 14 was calculated using the 2^−∆∆CT^ method from averages of three samples for each group. Human GAPDH (Hs_GAPDH_1_SG) was used as the housekeeping gene.

### 2.6. Proteome Profiling of Secreted Factors from Healthy and Diabetic ADSCs on SDF-1α Gene-Activated Scaffold

To understand how the SDF-1α gene-activated scaffold affected the production of therapeutic factors in diabetic ADSCs, we adopted the similar profiling method described in Section 2.4. The secretory profile of the diabetic ADSCs was again compared to that of the healthy ADSCs on the gene-free scaffold and on the gene-activated scaffold.

### 2.7. Pro-Angiogenic Bioactivity Analyses of Secreted Factors from the ADSCs on SDF-1α Gene-Activated Scaffold

Next, to determine the angiogenic impact of secreted factors, human umbilical vein endothelial cells (HUVECs, Cat no. 10HU-012, iXcells Biotechnologies) were exposed to CM collected from the ADSCs’ culture on day 7, and the subsequent angiogenic response by HUVECs in terms of network branching and tubule formation on Matrigel^TM^ (Corning, UK) was assessed. The HUVECs were seeded at a density of 3 × 10^4^ cells/well of a 48-well plate pre-coated with 120 μL of Matrigel for 30 min at 37 °C. The angiogenic response was monitored at 4, 8 and 24 h post-exposure to CM. At 8 h, the mean number of branching points and tubules was counted using the ImageJ software (ImageJ, NIH, Bethesda, MD, USA). All the images were captured at 10× magnification using an IX73 (Olympus, Tokyo, Japan) inverted microscope.

### 2.8. Immunofluorescent Imaging

Immunofluorescence staining was performed to detect the expression of target proteins by ADSCs. Scaffolds harvested at days 7 and 14 were used for the study. The scaffolds were first washed with PBS and fixed in 10% neutral buffered formalin for 20 min. The fixed samples were then processed using the standard protocol for paraffinization. The blocks were then cut into 8-μm thick slices and collected on charged slides. The sections were then deparaffinized using xylene followed by rehydration of the section with decreasing gradients of ethanol. Subsequently, the cells were permeabilized with 0.2% Tween^®^20 (Sigma-Aldrich, Saint-Quentin-Fallavier, France) solution in PBS for 30 min (10 min wash × 3) and blocked using 10% NGS (Normal Goat Serum, Invitrogen, Renfrew, UK)/5% BSA/0.3M Glycine (prepared in permeabilizing solution) for 1 h. The slides were briefly rinsed in PBS and then incubated at 4 °C overnight with antibodies against SDF-1α (rabbit mAb, 1:100, ab155090), CXCR7 (mouse mAb, 1:50, MAB42273), fibronectin (rabbit polyAb, 1:100, ab2413) and collagen IV (rabbit polyAb, 1:100, ab6586). All the primary antibodies were obtained from Abcam UK, except CXCR7 (R&D systems, Abingdon, UK).

The next day, the slides were rinsed in PBS thrice for 2–3 min each to remove any unbound primary antibodies. Subsequently, the slides were incubated in either Alexa 488-conjugated goat anti-mouse IgG (A32723, Invitrogen, UK) or Alexa 594-conjugated goat anti-rabbit IgG (A11012, Invitrogen, UK) at 1:800 dilution at room temperature for 1 h in the dark. The rinsing step was performed as before and counterstained for nuclei using the mounting medium with DAPI (ab104139, Abcam, UK). The slides were then imaged using a fluorescence microscope (Olympus BX43, Japan) at 40× objective. Samples incubated with only secondary antibodies were used as controls. All the antibodies were diluted in 1% BSA in PBS.

### 2.9. Image Analysis

The “ImageJ” software (ImageJ, NIH, Bethesda, MD, USA) was used to semi-quantitatively determine the amount of expressed proteins. For each marker, a constant threshold value was first determined through preliminary imaging of various sections. Using the set threshold value, integrated density (stained area x mean gray value) of the images was determined and then normalized to the number of cells (nuclei counting) to give a final mean fluorescence density per cell. An average was quantified from 8–12 random non-overlapping images per replicate, with a minimum of 3 replicates per group. The averages obtained from the 3 replicates/group were then used for measuring relative expression between the groups.

### 2.10. Statistical Analysis

All results are expressed as mean ± standard deviation. An unpaired two-tailed t-test was used to demonstrate the statistical significance between groups, where *p* < 0.05 was considered to be significant.

## 3. Results

### 3.1. Diabetes Impairs Signaling of Functional Factors in Human ADSCs

Figure 1B shows the relative expression of the functional factors secreted by the ADSCs on day 7. Overall, the diabetic ADSCs showed elevated production of inflammatory cytokine IL-8 (2-fold), anti-angiogenic factors PAI-1 (1.8-fold), TIMP-1 (2.9-fold), PEDF (2.2-fold) and TSP-1 (2.2-fold) and vascular destabilizing factor Ang-2 (1.8-fold) relative to their healthy equivalent. Relatively, the diabetic ADSCs also showed reduced production of MCP-1 by 40%. Meanwhile, the levels of pro-angiogenic factors (VEGF, ANG and Ang-1) between the two groups were comparable. Therefore, taken together, this finding verifies that functional signaling is impaired in diabetic ADSCs. Table 1 provides the functional role of the secreted factors in wound healing.

### 3.2. SDF-1α Gene-Activated Scaffold Promotes Overexpression of SDF-1α mRNA and Engages the CXCR7/β-Arrestin Signaling in Diabetic ADSCs

**Figure 2:**
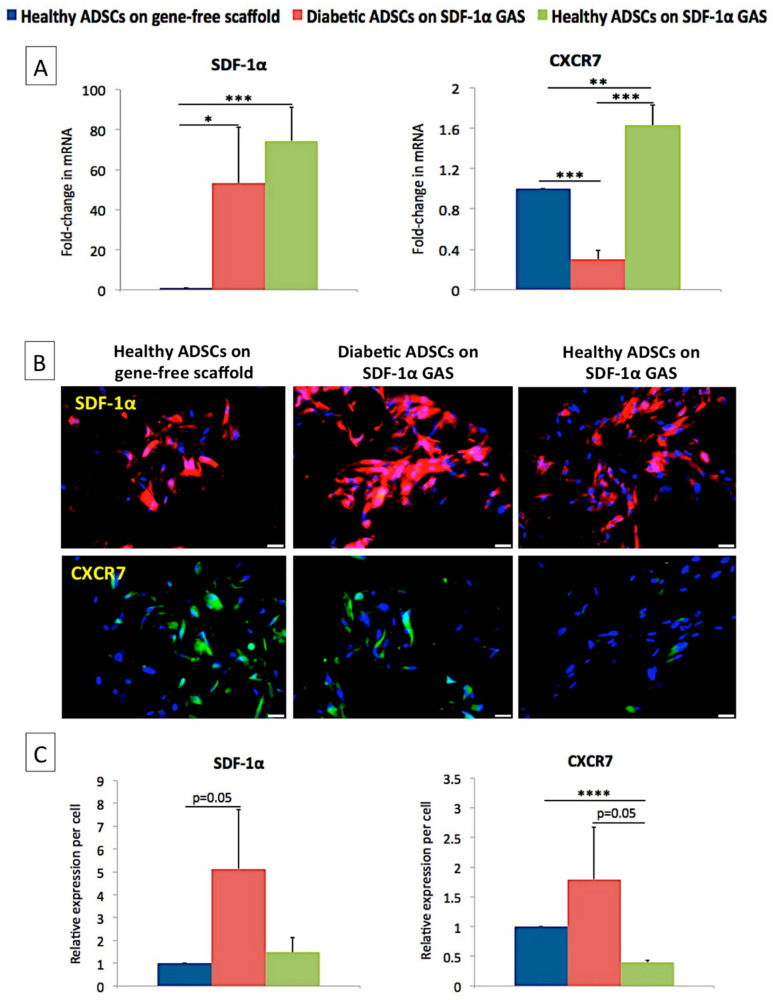
Impact of SDF-1α gene-activated scaffold on the activation of SDF-1α and its downstream signaling mediators in diabetic ADSCs. (**A**) SDF-1α gene-activated scaffold caused the overexpression of SDF-1α mRNA in both healthy and diabetic ADSCs. The overexpression of SDF-1α mRNA had a minimal effect on the expression of CXCR7 mRNA in diabetic ADSCs, while it increased significantly in healthy ADSCs. (**B**) Immunofluorescence images showing the abundancy of SDF-1α and CXCR7 in the ADSCs groups. (**C**) The diabetic ADSCs on the SDF-1α gene-activated scaffold expressed the highest level of SDF-1α and CXCR7 proteins, while healthy ADSCs displayed the weakest expression of CXCR7. *, **, *** and **** indicate statistical significance at *p* < 0.05, *p* < 0.01, *p* < 0.005 and *p* < 0.001, respectively. Data are presented as mean ± standard deviation (*n* = 3). Scale bar 20 μm.

Gene expression analysis first showed that the SDF-1α gene-activated scaffold caused the overexpression of SDF-1α mRNA in diabetic ADSCs, similar to the transfected healthy ADSCs. Having observed this, we then looked for the receptors associated with SDF-1α signaling. Of the two primary receptors for SDF-1α—CXCR4 and CXCR7—we could only detect the expression of CXCR7 mRNA (Figure 2). The healthy ADSCs on the gene-activated scaffold demonstrated significantly (*p* < 0.01) higher (63%) expression of CXCR7 mRNA than their gene-free scaffold equivalent. However, the diabetic ADSCs, despite overexpressing SDF-1α mRNA, showed a significantly (*p* < 0.005) lower (70%) activation of CXCR7 mRNA than the healthy ADSCs on the gene-free scaffold.

Immunofluorescence imaging then showed that all the ADSCs abundantly expressed SDF-1α proteins; however, contrary to the gene expression data, no obvious differences in the expression were observed. The diabetic ADSCs on the gene-activated scaffold showed the highest SDF-1α expression, while the expression of CXCR7 was comparable to that of the healthy ADSCs on the gene-free scaffold. The transfected healthy ADSCs, on the contrary, showed a significantly lower (*p* < 0.001; 61%) expression of CXCR7 than their gene-free scaffold equivalent.

Next, we assessed the activation of β-arrestin, which is the major downstream signal transducer of the SDF-1α/CXCR7 axis [71]. We found that the overexpression of SDF-1α in both healthy and diabetic ADSCs significantly enhanced the expression of β-arrestin mRNA (1.91 ± 0.33 for healthy; 1.88 ± 0.5 for diabetic) (*p* < 0.05) compared to that of the healthy ADSCs on the gene-free scaffold.

### 3.3. SDF-1α Gene-Activated Scaffold Restores a Healthy-Like Signaling of Functional Factors in Diabetic ADSCs

**Figure 3:**
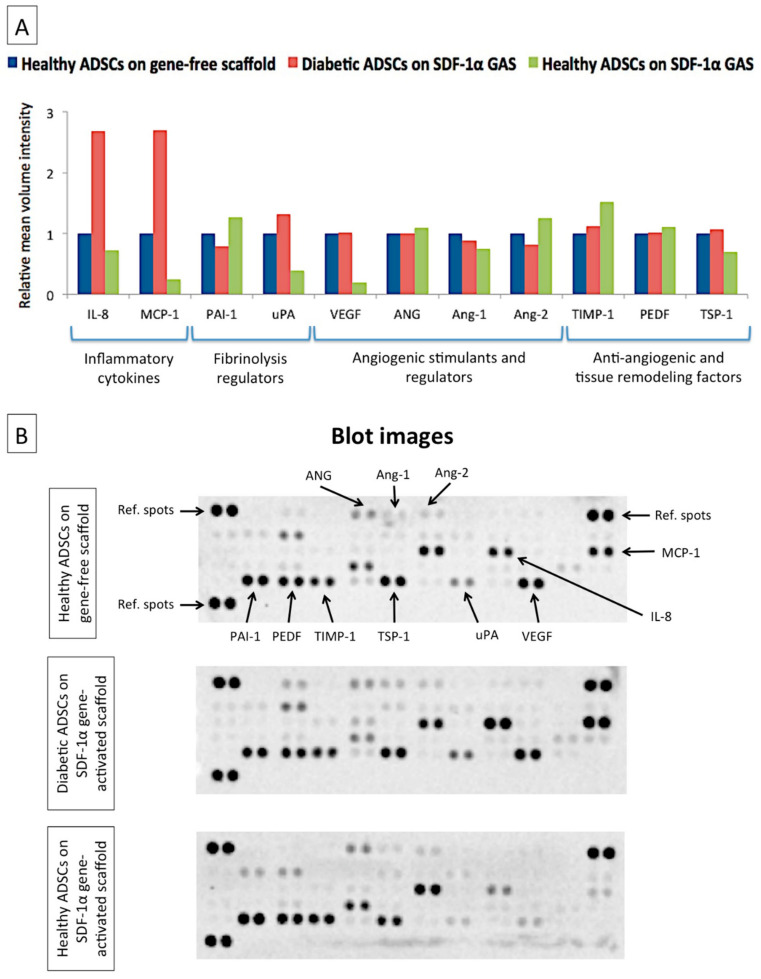
Impact of SDF-1α gene-activated scaffold on the production of functional factors in healthy and diabetic ADSCs. (**A**) Transfection of the diabetic ADSCs within the SDF-1α gene-activated scaffold resulted in restoration of a healthy-like signaling of functional factors in the diabetic ADSCs. On the other hand, the SDF-1α gene-activated scaffold caused a moderate deviation in the signaling pattern of the functional factors in the healthy ADSCs relative to its unactivated equivalent. (**B**) Representative blot images of the proteome analysis.

Having determined that the SDF-1α gene-activated scaffold could effectively engage the SDF-1α/CXCR7 axis in the diabetic ADSCs, we then assessed if the SDF-1α signaling enhances the diabetic ADSCs’ functionality. Subsequently, we used proteome profiling to determine the functional activation of diabetic ADSCs. We noted that the diabetic ADSCs on the gene-activated scaffold showed markedly improved signaling of the functional factors, whose pattern closely resembled that of the healthy ADSCs on the gene-free scaffold (Figure 3A). However, the transfected diabetic ADSCs did not downregulate the production of its inflammatory cytokine IL-8. The activated diabetic ADSCs instead showed enhanced production of MCP-1 to a level comparable to IL-8 (Figure 3B).

On the other hand, the healthy ADSCs on the gene-activated scaffold showed a markedly reduced production of uPA, MCP-1 and VEGF. The transfected healthy ADSCs also showed a moderate shift in the pattern of production of anti-angiogenic factors. For instance, the production of TIMP-1 increased by 51%, while the production of TSP-1 decreased by 31% relative to that produced by their gene-free scaffold equivalent.

### 3.4. Diabetic ADSCs on the SDF-1α Gene-Activated Scaffold Effectively Enhance Angiogenesis in Endothelial Cells

**Figure 4:**
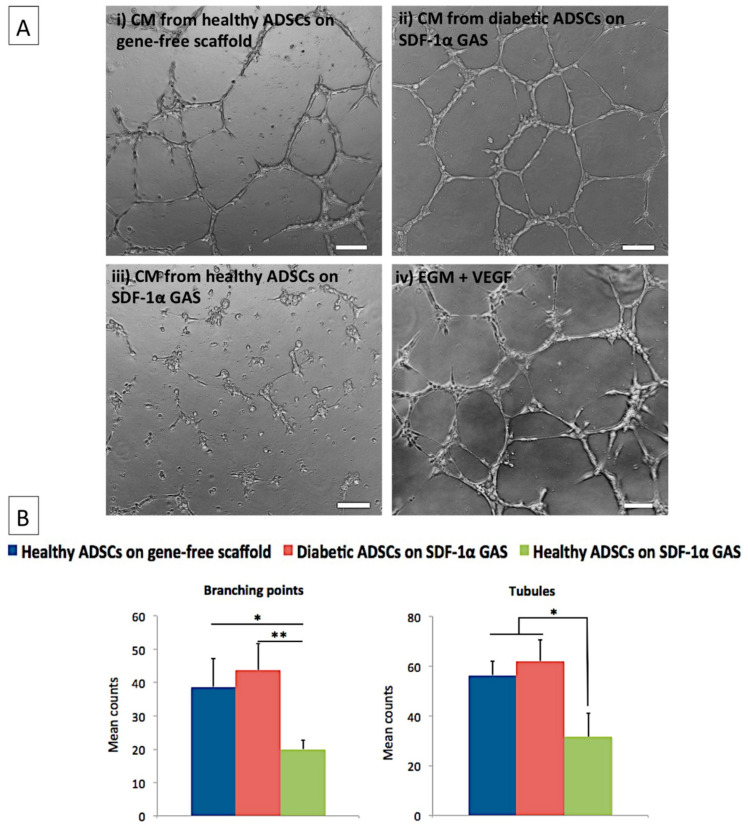
Pro-angiogenic impact of secreted factors from the transfected healthy and diabetic ADSCs. (**A**) The diabetic ADSCs on the SDF-1α gene-activated scaffold induced the strongest pro-angiogenic response in human endothelial cells. (**B**) At 8 h post-exposure, CM from diabetic ADSCs significantly enhanced endothelial network branching (*p* < 0.01) as well as tubules formation (*p* < 0.05), compared to that induced by their healthy counterpart. EndoGro Media (EGM) + VEGF was used as reference medium to stimulate endothelial angiogenesis. Scale bar 100 μm. * *p* < 0.01, ** *p* < 0.05.

One of the significant challenges in the healing of diabetic wounds is the lack of angiogenesis [72]. Therefore, having noted an enhanced pro-angiogenic profile in transfected diabetic ADSCs, we assessed the pro-angiogenic bioactivity of the secreted factors on endothelial cells. Treatment of the endothelial cells with CM from the transfected diabetic ADSCs promoted the formation of well-defined endothelial tubular networks by 8 h post-treatment (Figure 4A ii). The mean number of branching points and tubules was 43 ± 8 and 62 ± 8, respectively. The angiogenic response was relatively milder (39 ± 9 branching points; 56 ± 6 tubules) when stimulated with CM from healthy ADSCs on the gene-free scaffold. The angiogenic response was further lower (20 ± 3 branching points; 32 ± 9 tubules) in the endothelial groups exposed to CM from the transfected healthy ADSCs. Comparatively, the diabetic ADSCs on the SDF-1α gene-activated scaffold demonstrated significantly (*p* < 0.05) superior pro-angiogenic potency than their healthy counterpart.

### 3.5. SDF-1α Gene-Activated Scaffold Promotes Pro-Wound Healing Matrix Remodeling Response in Diabetic ADSCs

**Figure 5:**
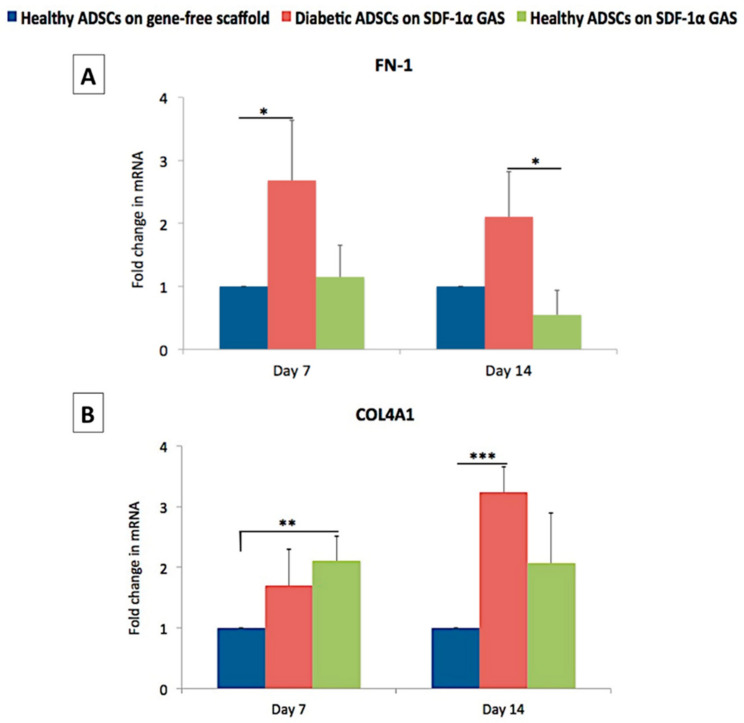
Effect of SDF-1α gene-activated scaffold on the expression of pro-wound healing matrix genes in healthy and diabetic ADSCs. (**A**) On day 7, the transfected diabetic ADSCs on the gene-activated scaffold showed a significantly (*p* < 0.05) enhanced transcription of the FN1 gene than the healthy ADSCs on the gene-free scaffold. (**B**) At the same time point, the healthy ADSCs on the gene-activated scaffold showed a significant activation of the COL4A1 gene than their gene-free equivalent. *, ** and *** indicate statistical significance at *p* < 0.05, *p* < 0.01 and *p* < 0.005, respectively. Data are presented as mean ± standard deviation (*n* = 3).

**Figure 6:**
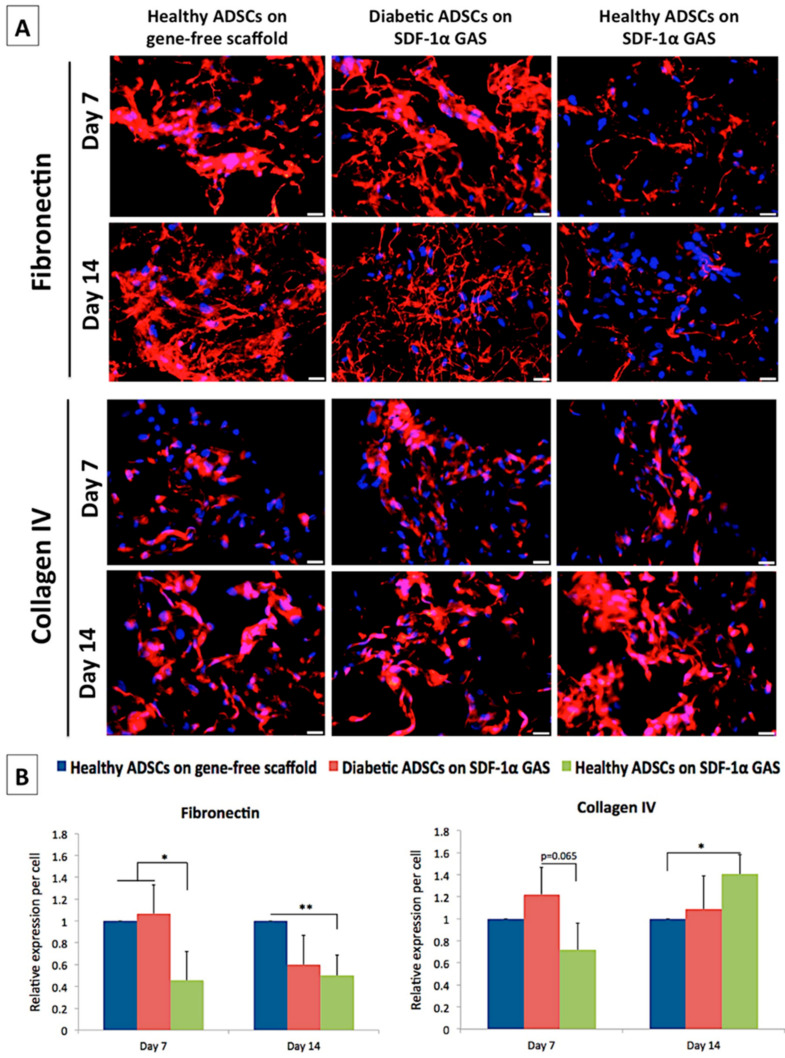
Effect of SDF-1α gene-activated scaffold on the deposition and remodeling of pro-wound healing matrix proteins in healthy and diabetic ADSCs. (**A**) Relative to the healthy ADSCs on the gene-free scaffold, diabetic ADSCs on the SDF-1α gene-activated scaffold showed a significant decrease in the deposition of fibronectin matrix while increasing the deposition of collagen IV over time. Contrarily, healthy ADSCs on the SDF-1α gene-activated scaffold deposited minimal amounts of the fibronectin matrix throughout the culture period but significantly increased the deposition of collagen IV. (**B**) Semi-quantitative interpretation of spatiotemporal expression of the matrix proteins. * and ** indicate statistical significance at *p* < 0.05 and *p* < 0.01, respectively. Data are presented as mean ± standard deviation (*n* = 3). Scale bar 20 μm.

Fibronectin is one of the first matrix proteins produced during the early stages of cellular development. It also acts as a provisional scaffold for subsequent matrix deposition [73]. Therefore, we first assessed the expression of the fibronectin gene and deposition of its matrix. On day 7, the diabetic ADSCs on the gene-activated scaffold showed a significantly enhanced transcription of the FN1 gene than the healthy ADSCs on the gene-free scaffold (Figure 5). Immunofluorescence analysis of matrix deposition further showed that the diabetic ADSCs abundantly deposited the matrix similar to that observed in the healthy ADSCs on the gene-free scaffold (Figure 6). However, on day 14, the fibronectin matrix remodeled into thin, continuous fibers, causing an overall reduction in the spatial coverage of 40% compared that of the healthy ADSCs on the gene-free scaffold. Conversely, the healthy ADSCs on the gene-activated scaffold did not show activation of the FN1 gene on day 7 but moderately downregulated its expression by 45% compared to that of their gene-free scaffold equivalent on day 14. At both time points, fibronectin deposition by the healthy ADSCs on the gene-activated scaffold was significantly (*p* < 0.05) lower than that on the gene-free scaffold equivalent (Figure 6B).

We next assessed the expression of collagen IV, which is essential for the formation of the basement membrane [74], a specialized structure that binds the dermis to the epidermis. Conversely to FN1 gene expression, we noted a significant (*p* < 0.01) early activation (day 7) of the COL4A1 gene only in the healthy ADSCs on the gene-activated scaffold. Furthermore, matrix deposition also increased significantly in the healthy ADSCs on the gene-activated scaffold compared to that on the gene-free scaffold equivalent. Meanwhile, the diabetic ADSCs on the gene-activated scaffold showed a temporal increase in the expression of the COL4A1 gene. On day 14, the expression of the COL4A1 gene by the diabetic ADSCs significantly (*p* < 0.005) exceeded that of the healthy ADSCs on the gene-free scaffold. However, at the protein level, the diabetic ADSCs on the gene-activated scaffold deposited comparable amounts of the collagen IV matrix to those of the healthy ADSCs on the gene-free scaffold.

**Figure 7:**
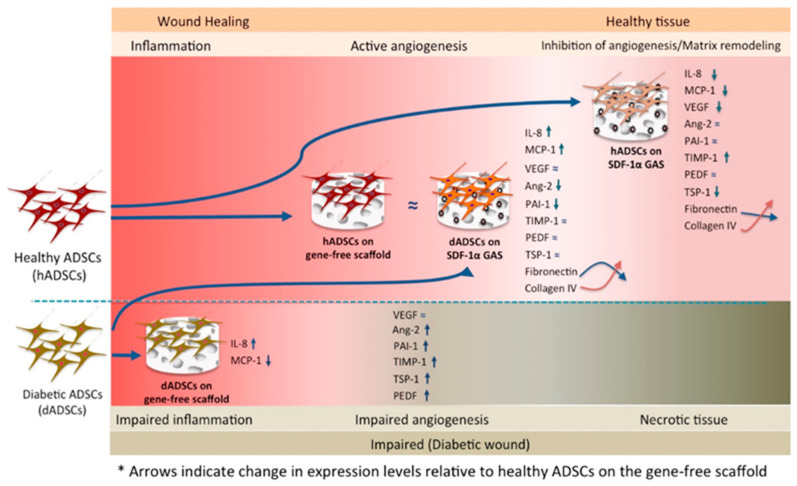
A schematic of the functional changes induced by the SDF-1α gene-activated collagen scaffold in healthy and diabetic ADSCs. The diabetic ADSCs on the gene-free scaffold initially showed an impaired functional response characterized by elevated inflammatory cytokine production (IL-8), anti-angiogenic factors (PAI-1, TIMP-1, PEDF and TSP-1) and vascular destabilizing factor Ang-2 compared to the healthy ADSCs on the gene-free scaffold. However, when activated within the gene-activated scaffold, the impaired signaling in the diabetic ADSCs could be restored to a healthy-like state and exert therapeutic paracrine effects capable of enhancing angiogenesis. Meanwhile, the gene-activated scaffold drove the healthy ADSCs towards an advanced cellular maturation stage while facilitating the bypass of early signaling events such as the production of uPA, VEGF and MCP-1 and the deposition of the provisional matrix fibronectin.

Taking the results together, we show that the SDF-1α gene-activated scaffold restores the pro-regenerative capacity in diabetic ADSCs similar to the healthy ADSCs on the gene-free scaffold. Further, we show that the SDF-1α gene-activated scaffold also promotes controlled development of healthy ADSCs towards a pro-healing nature. Figure 7 depicts the overall findings of this study.

## 4. Discussion

In this study, we explored the therapeutic impact of an SDF-1α gene-activated collagen scaffold on human diabetic ADSCs as an approach to develop a functional three-dimensional autologous graft for diabetic wound healing. We found that the SDF-1α gene-activated scaffold restored pro-angiogenic signaling in the diabetic ADSCs similar to the healthy ADSCs on the gene-free scaffold. The transfected diabetic ADSCs also exhibited active matrix remodeling events characterized by a reduction in the deposition of the fibronectin matrix and an increase in the expression of basement membrane protein collagen IV. Meanwhile, in healthy ADSCs, the SDF-1α gene-activated scaffold promoted controlled cellular maturation, by instructing the ADSCs to disable the signaling of early developmental factors and promote the production of tissue remodeling components crucial for wound healing.

The impaired healing response in diabetic patients is attributed to the reduced regenerative potential of the endogenous cells [46]. Diabetic ADSCs often show reduced expression of pro-angiogenic factors, and this is considered a significant factor limiting their application for cellular therapy [18,21,75,76]. Nevertheless, wound healing is a complex process controlled by a cocktail of signaling factors including anti-angiogenic factors [77]. Therefore, we used an angiogenesis proteome profiler that enabled us to differentiate between the multiple signaling factors produced by the healthy and diabetic ADSCs when grown in a three-dimensional scaffold. We noted that it is not necessarily the lack of a pro-angiogenic component but an impaired production of anti-angiogenic and vascular destabilizing factors that may also limit the functional potency of ADSCs (Figure 1). Other studies have also found the association of elevated levels of anti-angiogenic factors such as PAI-1 [78] and PEDF [79] with poor healing response in diabetic wounds. An imbalanced ratio of Ang-2 with Ang-1 has also been implicated as a predictor of poor healing outcomes in diabetic wounds [80,81].

Subsequently, we sought to assess if the SDF-1α gene-activated scaffold could restore the diabetic ADSCs’ impairment to an improved functional state. As anticipated, we noted that SDF-1α gene delivery using the gene-activated scaffold restores the impairment in secretome production in the diabetic ADSCs as in healthy ADSCs on the gene-free scaffold (Figure 3). Stem cells’ secretome is a key component for driving wound healing by instructing surrounding cells such as the endothelial cells and modulating the wound environment [82]. Therefore, the resulting normalization of the diabetic ADSCs induced by the gene-activated scaffold may offer enhanced healing capabilities in autologous treatment strategies. We also proved, in vitro, that the secreted factors from the diabetic ADSCs on the gene-activated scaffold can stimulate angiogenic growth in endothelial cells at a similar capacity as that of the healthy ADSCs on the gene-free scaffold (Figure 4). Moreover, in tissue engineering strategies, a construct’s ability to induce an enhanced angiogenic response is crucial for faster integration of the graft with the host environment [83].

Among the secreted factors, a notable response induced by the gene-activated scaffold in the diabetic ADSCs is the elevated production of MCP-1. In diabetic wounds, the lack of MCP-1 is considered a major factor halting the progression of healing [84,85]. Wood et al. showed that early treatment with MCP-1 at the time of injury significantly enhanced macrophage infiltration, thereby accelerating healing in diabetic mice [84]. Investigations on healthy wounds also found that the elevation of MCP-1 sequential to IL-8 is crucial to drive an acute-like healing [53]. Therefore, despite the production of high levels of IL-8, the ability of the transfected diabetic ADSCs to equilibrate the level of MCP-1 to IL-8 may facilitate a timely transition to the subsequent healing phase.

While the SDF-1α gene-activated scaffold works to improve angiogenic homeostasis in diabetic ADSCs, it appears to disable the signaling components of the early healing phase in healthy ADSCs. For instance, in healthy ADSCs, the SDF-1α gene-activated scaffold robustly dampened the production of pro-inflammatory MCP-1 and pro-angiogenic VEGF. However, it did not compromise the signaling of the anti-angiogenic tissue inhibitors, which are essential for tissue remodeling [57,67,68,70]. It has been observed that stem cells possess a unique ability to sense external stimuli and accordingly modulate their secretome to offer a cytoprotective effect [86]. In our study, the modulatory impact of the SDF-1α gene-activated scaffold in the ADSCs appears to be controlled by the receptor CXCR7 (Figure 2C). A recent study that investigated the impact of SDF-1α on the differentiation potential of embryonic stem cells (ESCs) showed that the presence of active or inactive CXCR7 differentially modulates the development of ESCs [87]. The group showed that the engagement of active CXCR7 by SDF-1α leads to downregulation of pluripotency markers without affecting the expression of factors essential for development in wild-type ESCs [87]. Conversely, in mutant cells with inactive CXCR7, the expression of the pluripotency markers was barely affected [87]. Similarly, given that diabetic ADSCs are relatively dysfunctional, the sensitivity of CXCR7 to SDF-1α is probably weaker, leading to a varied response than transfected healthy ADSCs. Nevertheless, activation of CXCR7 is known to promote survival and proliferation of ADSCs [17].

Since CXCR4 is the classical receptor of SDF-1α [17,88], we anticipated that the overexpression of SDF-1α induced by the gene-activated scaffold would upregulate the expression of CXCR4 in the transfected ADSCs. However, we barely detected the expression of CXCR4 mRNAs even in the untransfected healthy ADSCs. A similar case has been observed in MSCs [89], but we cannot rule out that the gene is completely degraded, as it would lead to cell death [90]. It has been observed that the CXCR4 gene is generally expressed at very low levels in human ADSCs and is also the least expressed among the set of chemokine receptor genes [91]. Another factor for the undetection of CXCR4 could be the activation of CXCR7. The activation of CXCR7 can dampen the expression of CXCR4 [92]. However, the mechanism that triggered the activation of CXCR7 over CXCR4 even in the untransfected ADSCs in the gene-free scaffold needs further investigation. Nevertheless, a study by Lisignoli et al. [93] suggests that the matrix may modulate the expression of chemokine receptors. They found that the transcriptional expression of SDF-1α and CXCR4 is reversed when the MSCs are cultured on a hyaluronic acid-based scaffold compared to a plastic substrate [93]. The MSCs on the hyaluronic acid scaffold showed downregulation of SDF-1α mRNA while upregulating CXCR4 mRNA relative to the MSCs on the plastic substrate [93]. The group found that the activation of CD54, a cell surface receptor of hyaluronan, on the MSCs modulated this response [93]. In our context, the ADSCs are known to express the collagen receptor α2β1 integrin [94]. The receptor α2β1 integrin promotes the adhesion and proliferation of stem cells on collagen [95]. Additionally, CXCR7 is essential for the promotion of proliferation and survivability in ADSCs [17,96]. Therefore, assuming the regulatory role of the matrix, it appears that a proliferative mechanism may have been activated upon initial adhesion, leading to the upregulation of CXCR7 but not CXCR4.

The adipose ECM is also known to possess pro-healing properties [97]. Therefore, we investigated how the SDF-1α gene-activated scaffold affects diabetic ADSCs in terms of matrix deposition and remodeling. We focused on the expression and deposition pattern of two of the traditional matrix proteins—the provisional matrix protein, fibronectin, and a relatively mature basement membrane protein, collagen IV. Fibronectin is not only essential for supporting the adhesion and migration of cells but also provides a scaffold for subsequent collagen deposition [73]. However, diabetic skin lacks this feature, and this is one of the causes that impede healing [98,99]. To improve healing, soluble forms of fibronectin are supplemented to the wound to facilitate the assembly of the fibronectin matrix and promote healing [100,101]. Here, we show that the SDF-1α gene-activated scaffold effectively enhances the deposition of the fibronectin matrix by the diabetic ADSCs, imparting the potential to drive healing. Furthermore, the response that the transfected diabetic ADSCs are driven towards healing could also be evident from their high transcriptional activity of the COL4A1 gene and assembly of the collagen IV matrix over time. The SDF-1α gene-activated scaffold also enhanced the deposition of collagen IV in the healthy ADSCs, suggesting that it promotes cellular maturation. However, the increase in collagen IV deposition by the transfected healthy ADSCs occurred despite bypassing the activation of either the FN1 gene or its assembly as a matrix. Collectively, these events imply that the SDF-1α gene-activated scaffold promotes controlled remodeling of ADSCs’ ECM to create a pro-healing environment regardless of the physiological state of the ADSCs. This controlled response in the ADSCs could be attributed to the homeostatic role of SDF-1α [102].

Specifically, this study addressed a potential solution for developing an enhanced autologous bioengineered graft using diabetic ADSCs, which are generally impaired. Patients requiring stem cell therapy could avoid the search for matching healthy donors and may use their own cells for the treatment. It may both reduce the time of treatment and costs [103]. However, this study’s limitation is that we did not investigate the activated diabetic ADSCs’ immunomodulatory effect on immune cells. ADSCs are known to exert immunosuppressive effects in an inflammatory environment [104]. Therefore, direct or indirect co-culture of immune cells with the activated diabetic ADSCs will help better understand the therapeutic implications of the gene-activated scaffold/ADSCs construct. Further studies also need to be performed to determine the product’s shelf life and potential adverse reactions in vivo, the details of which are crucial for successful implementation in a hospital setting [105]. Although not approved by the FDA yet, the gene-activated scaffold components, i.e., the vector PEI [106] and the SDF-1α plasmid [107], have been safely used in clinical trials. Therefore, we anticipate that further functional studies using in vivo models will help in more quickly translating the technology to clinics.

## 5. Conclusions

In this study, we report that a three-dimensional collagen–chondroitin sulfate scaffold functionalized with a pro-angiogenic SDF-1α gene could be used to enhance the functionality of human diabetic ADSCs as effectively as healthy ADSCs on a gene-free scaffold. Specifically, overexpression of SDF-1α in the diabetic ADSCs led to normalization of the production of therapeutic factors, restoring their pro-angiogenic potency. The diabetic ADSCs also exhibited a pro-healing feature characterized by active matrix remodeling of fibronectin and collagen IV matrixes. We also note that it is not necessarily the lack of a pro-angiogenic component but an impaired production of anti-angiogenic and vascular destabilizing factors that may also limit the functional potency of diabetic ADSCs. Conclusively, we have shown that a pro-angiogenic collagen biomaterial can enhance the wound healing response of typically dysfunctional diabetic ADSCs, paving the way for better patient-specific DFU treatment.

## Figures and Tables

**Table 1:** ADSCs’ secreted factors and their role in angiogenic wound healing.

Soluble Factor	Full Name	Function
IL-8	Interleukin-8	Recruitment of neutrophils; component of early inflammatory phase; angiogenic [53,54]
MCP-1	Monocyte chemoattractant protein-1	Recruitment of monocytes; essential for progression of healing; angiogenic [53,55]
PAI-1	Plasminogen activator inhibitor-1	Serine protease inhibitor; inhibits fibrinolysis; promotes re-epithelialization; anti-angiogenic [56,57]
uPA	Urokinase-type plasminogen activator	Mediates fibrinolysis; promotes angiogenesis [58,59]
VEGF	Vascular endothelial growth factor	Pro-angiogenic factor; stimulates vessel sprouting [60]
ANG	Angiogenin	Exerts ribonuclease activity; regulates VEGF-induced endothelial proliferation and angiogenesis [61]
Ang-2	Angiopoietin-2	Promotes vessel sprouting with VEGF; exerts vascular destabilizing effects [62,63]
Ang-1	Angiopoietin-1	Exerts vascular protective effects; essential for vessel stabilization [64]
TIMP-1	Tissue inhibitor of metalloproteinase-1	Anti-angiogenic; involved in the regeneration of epidermis and matrix remodeling [65,66,67]
PEDF	Pigment epithelium-derived factor	Potent anti-angiogenic factor; expression increases at late stage of wound healing; promotes resolution of angiogenesis [68]
TSP-1	Thrombospondin-1	Matricellular protein; inhibits angiogenesis; elevated expression suppresses wound healing [69,70]

## Data Availability

The datasets used and/or analyzed during the current study are available from the corresponding author on reasonable request.

## List of Abbreviations

ADSCs	Adipose-derived stem cells
Ang-2	Angiopoeitin-2
BM-MSCs	Bone marrow-mesenchymal stem cells
CSF	Colony stimulating factor
Coll–CS	Collagen–chondroitin sulfate
CM	Conditioned media
ECM	Extracellular matrix
EDAC/NHS	N-(3-Dimethylaminopropyl)-N’-ethylcarbodiimide hydrochloride/N-Hydroxysuccinimide
FDA	Food and Drug Administration
GAS	Gene-activated scaffold
HUVECs	Human umbilical vein endothelial cells
IGFBP-3	Insulin growth factor binding protein-3
IL-8	Interleukin-8
MCP-1	Monocyte chemoattractant protein-1
PAI-1	Plasminogen activator inhibitor-1
pDNA	Plasmid DNA
PEI	Polyethyleneimine
PEDF	Pigment epithelium-derived factor
SDF-1α	Stromal-derived factor-1 alpha
TIMP-1	Tissue inhibitor of metalloproteinase-1
TSP-1	Thrombospondin-1
VEGF	Vascular endothelial growth factor
